# Documentation of Driving Status and Driving Advice for Adult Mental Health Inpatients: A Quality Improvement Project

**DOI:** 10.1192/bjo.2026.11365

**Published:** 2026-06-30

**Authors:** Rhiannon Coulter, Emma Norris, Aidan Turkington

**Affiliations:** Belfast Health and Social Care Trust, Belfast, United Kingdom

## Abstract

**Aims:**

The aim of this quality improvement project was to improve the documentation of driving status and, where relevant, driving advice in patients’ electronic record during admission in one acute adult mental health inpatient unit by 10% within 8 weeks.

**Methods:**

Two outcome measures were established: (A) Driving status documented in notes and/or discharge letter, (B) Driving advice for drivers documented in notes and/or discharge letter.

The electronic notes and discharge letters of patients discharged from the unit each week were retrospectively audited and weekly proportions of each measure calculated.

Using Plan, Do, Study, Act cycles, three interventions were implemented sequentially: (1) Education session delivered to resident doctors on driving regulations for psychiatric disorders; (2) Displaying a poster prompt in the resident doctors’ communal workspace; and (3) Addition of a mandatory field for driving status check to the standardised discharge meeting template.

Data was analysed using run charts with baseline and post-intervention medians.

**Results:**

Retrospective audit of 164 patients’ records was completed, with 85 patients from the 8-week baseline period and 79 from the 9-week intervention period.

In the baseline period, 48 patients had driving status documented. In the intervention period, 53 patients had driving status documented. The median documentation of driving status improved from 60% to 83%. There was a shift on run chart analysis with all 9 data points in the intervention period remaining above the baseline median.

Driving advice was documented in 10 out of 20 drivers during the baseline period and 17 out of 27 in the intervention period. The median of documented driving advice given to known drivers improved from 50% at baseline to 80% post-intervention. However, there was substantial week-to-week variability and multiple weeks of zero compliance.

**Conclusion:**

The results indicate that the three interventions produced sustained improvement in documentation of driving status, reflecting system change, and meeting the aim of this project. These three simple interventions could be replicated quickly and at low cost to other acute inpatient psychiatric units to spread this improvement beyond the single unit involved in this project.

Although the median of documented driving advice to drivers improved, to meet the aim of the project, the lack of shift on run chart analysis reflects that the interventions did not create sustainable system change. Further work is required to ensure patients who drive have been given the appropriate advice and had it documented in their electronic record, prior to discharge from this acute adult mental health inpatient unit.

